# Helminths in Wild Canids from Three Brazilian Biomes: Parasitological and Pathological Records

**DOI:** 10.3390/biology15201875

**Published:** 2026-10-17

**Authors:** Zara Mariana de Assis-Silva, Iago de Sá Moraes, Bruna Samara Alves-Ribeiro, Lizandra Fernandes-Silva, Ana Paula Carvalho Gomes, João Victor de Oliveira Araújo Alves, Nicoly Ferreira de Urzedo, Klaus Casaro Saturnino, Ísis Assis Braga, Ana Paula Nascimento Gomes, Renan Mendes Pires Moreira, Beatriz Elise de Andrade-Silva, Maria Isabel Afonso da Silva, Tiago Lucena da Silva, Estevam Guilherme Lux Hoppe, Arnaldo Maldonado Júnior, Edson Moleta Colodel, Richard de Campos Pacheco, Dirceu Guilherme de Souza Ramos

**Affiliations:** 1 Laboratório de Parasitologia Veterinária e Doenças Parasitárias dos Animais Domésticos e Silvestres, Faculdade de Medicina Veterinária, Universidade Federal de Mato Grosso, Cuiabá 78060-900, Brazil; 2 Laboratório de Anatomia Patológica Veterinária, Instituto de Ciências Agrárias, Universidade Federal de Jataí, Jataí 75801-615, Brazilklaus.sat@ufj.edu.br (K.C.S.); 3 Laboratório de Parasitologia Veterinária, Instituto de Ciências Agrárias, Universidade Federal de Jataí, Jataí 75801-615, Brazilanapc_gomes@discente.ufj.edu.br (A.P.C.G.);; 4 Laboratório Integrado de Análises Clínicas e Infectologia Veterinária, Instituto de Ciências Agrárias, Universidade Federal de Jataí, Jataí 75801-615, Brazil; 5 Laboratório de Helmintos Parasitos de Vertebrados, Instituto Oswaldo Cruz, Fiocruz, Rio de Janeiro 21040-900, Brazil; 6 Laboratório de Helmintos Parasitos de Peixes, Instituto Oswaldo Cruz, Fiocruz, Rio de Janeiro 21040-900, Brazil; 7 Laboratório de Biologia Animal, Campus Floresta, Universidade Federal do Acre, Cruzeiro do Sul 69895-000, Brazil; maria.afonso@ufac.br (M.I.A.d.S.);; 8 Laboratório de Enfermidades Parasitárias e Zoonoses, Faculdade de Ciências Agrárias e Veterinárias, Campus Jaboticabal, Universidade Estadual Paulista Júlio de Mesquita Filho, Jaboticabal 14884-900, Brazil; 9 Laboratório de Biologia e Parasitologia de Mamíferos Reservatórios, Instituto Oswaldo Cruz, Fiocruz, Rio de Janeiro 21040-900, Brazil; 10 Laboratório de Patologia Veterinária, Faculdade de Medicina Veterinária, Universidade Federal de Mato Grosso, Cuiabá 78060-900, Brazil; edson.colodel@ufmt.br

**Keywords:** carnivora, one health, neotropical, wildlife, zoonotic helminths

## Abstract

Wild canids inhabiting anthropized landscapes can act as environmental indicators and potential reservoirs of zoonotic parasites, connecting transmission cycles among wild, domestic, and human hosts. We aimed to identify and characterize helminths in wild canids from the Cerrado, Pantanal, and Amazon biomes of Brazil, describing occurrence patterns and ecological and One Health implications. A total of 19 individuals belonging to four wild canid species (*Cerdocyon thous*, *Chrysocyon brachyurus*, *Lycalopex vetulus*, and *Speothos venaticus*) were examined, yielding 1041 helminths representing 17 taxa, with nematodes predominating. Several taxa of recognized zoonotic importance were recovered, including *Toxocara canis*, *Ancylostoma* spp., *Dioctophyme renale*, and *Spirometra* spp. These are agents associated with visceral and cutaneous larva migrans, dioctophymosis, and sparganosis. Novel host records were documented, including *Athesmia foxi* and *Pterygodermatites affinis* in *L. vetulus*, a sparganum infection in *C. thous*, and the first report of *Spirocerca lupi* in the stomach of *S. venaticus* in the Brazilian Cerrado. These findings expand the knowledge of the helminth fauna of Neotropical wild canids and reinforce the relevance of parasitological surveillance in wildlife as a tool for zoonotic disease monitoring, biodiversity conservation, and environmental health assessment, reaffirming the role of wild canids as ecological indicators.

## Simple Summary

Parasitism in wild animals is an important bioindicator of ecosystem integrity. Wild canids, such as the crab-eating fox, maned wolf, and bush dog, inhabit natural areas subject to increasing human interference and can serve as indicators of parasite occurrence in the environment. In this study, researchers examined 19 wild canids of four species across three biomes of Brazil (Cerrado, Pantanal, and Amazon) for helminths. Seventeen parasite species, totaling 1041 worms, predominantly nematodes, were identified. Some of these parasites can also infect humans and domestic animals, causing diseases such as larva migrans (cutaneous or visceral), dioctophymosis, and sparganosis. The study yielded novel findings, including new host–parasite interactions for wild canid species. These results reinforce the importance of monitoring parasites in wild animals to survey diseases that may affect humans and domestic animals, protect biodiversity, and assess the health of natural environments.

## 1. Introduction

Parasitism in wild animals is an important bioindicator of ecosystem integrity, enabling the assessment of pathogen circulation and ecological changes associated with environmental alterations and host population dynamics [1]. Wildlife serves as an important epidemiological reservoir of parasitic zoonoses, with potential impact at the animal, environmental, and human health interface, particularly in contexts of increasing anthropization and fragmentation of natural habitats, which favor spillover events [2].

Among wild mammals, canids are particularly important as environmental indicators and potential reservoirs of a wide range of parasites, including species shared between domestic animals and humans, owing to their generalist diet and trophic interactions [3,4]. Currently, 35 species of wild canids are recognized worldwide, six of which occur in Brazil, distributed across five genera [5].

According to the United Nations (ONU, 2019), Brazil is one of the world’s megadiverse countries and harbors approximately 20% of the world’s biota, encompassing a diverse array of ecosystems and wildlife species. Noteworthy biomes include the Cerrado, classified as one of the global biodiversity hotspots, with high endemism rates associated with intense anthropogenic pressures; the Amazon, which concentrates one of the greatest diversities of endemic flora and fauna on the planet; and the Pantanal, the largest continental floodplain, recognized for its remarkable biological richness [6,7,8].

Despite their ecological significance, these biomes face increasing pressure from anthropogenic factors, including agricultural frontier expansion, urban growth, pollution, illegal hunting and capture of wildlife, and unnatural wildfires and forest fires [9]. The loss and fragmentation of vegetation cover compromise landscape connectivity, restricting the movement of native fauna and increasing their vulnerability to anthropogenic interference [10]. Such changes result in reduced resource availability, a decline in taxonomic and functional diversity, and modifications in the ecological processes that regulate disease transmission dynamics [11,12].

Parasitic infections, particularly soil-transmitted helminthiases, are among the most prevalent worldwide, representing a significant risk to human and animal health. Gastrointestinal parasites of canids, including helminths of recognized public health importance, such as those in the genera *Ancylostoma*, *Echinococcus*, *Toxocara*, and *Trichuris*, are of epidemiological relevance owing to environmental contamination with feces containing infective stages [13,14].

Parasites are common components of wildlife, and although they may appear to be a secondary factor in the conservation of wild species, evidence demonstrates that they can directly influence the abundance, distribution, and viability of natural populations [15]. Therefore, understanding parasite diversity contributes to comprehending the ecological processes that shape biodiversity [16].

We aimed to identify helminths in wild canids from the Cerrado, Pantanal, and Amazon biomes. These data contribute to understanding how anthropization influences the risks that these parasites pose to animal, environmental, and human health.

## 2. Materials and Methods

**Figure 1:**
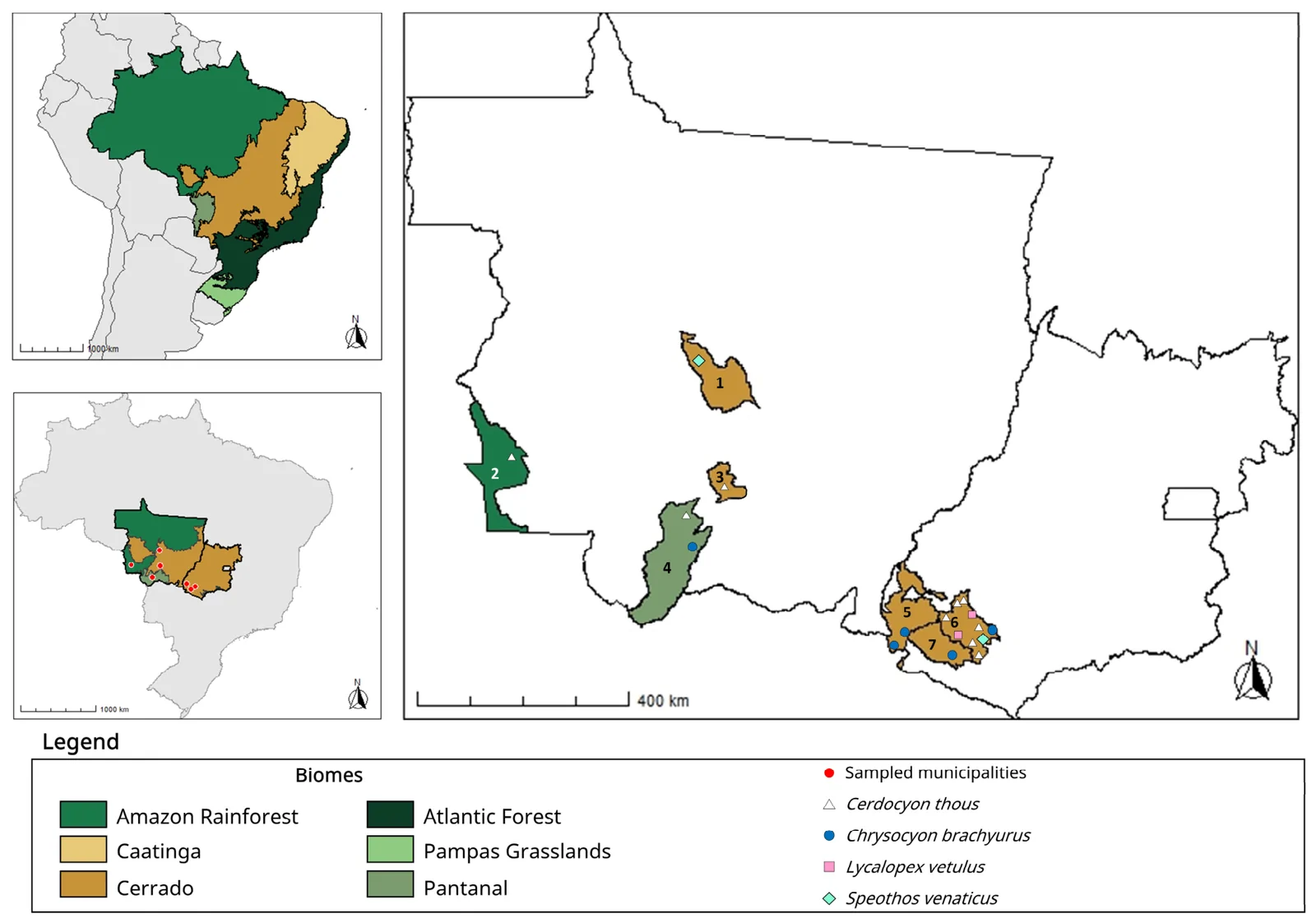
Geographic distribution of collection sites and biomes of wild canid carcasses in Brazil (R Studio 26.10.0, R Development Core Team). Municipalities: 1—Nova Mutum; 2—Vila Bela da Santíssima Trindade; 3—Cuiabá; 4—Poconé; 5—Mineiros; 6—Jataí; 7—Serranópolis.

The study was conducted in areas encompassing three biomes: the Cerrado, in the states of Mato Grosso and Goiás, and the Pantanal and Amazon, both in the state of Mato Grosso (Figure 1). Given that some of the studied species (*Chrysocyon brachyurus*, *Speothos venaticus*, and *Lycalopex vetulus*) are listed as Vulnerable in the Brazilian National Action Plan for the Conservation of Wild Canids [17], the sampling design was based on the opportunistic collection of wild canid carcasses. The animals were obtained from peri-urban roads in southwestern Goiás following vehicle collisions, or died in the Veterinary Hospital of the Federal University of Mato Grosso (UFMT) during clinical care.

A total of 19 wild canid specimens were examined, comprising 10 *Cerdocyon thous* (Crab-eating Fox), 5 *C. brachyurus* (Maned Wolf), 2 *L. vetulus* (Hoary Fox), and 2 *S. venaticus* (Bush Dog). All animals were classified as adults based on morphological criteria, including dentition wear pattern, reproductive organ development, and average body size for the species. As most carcasses originated from roadkill events, sex and biometric data (e.g., body weight and length) were not consistently available for all specimens and were therefore not included in the parasitological analysis. The carcasses were subjected to complete parasitological necropsy. All body cavities (thoracic, abdominal, and pericardial), internal organs (lungs, liver, kidneys, urinary bladder, and heart), and the entire gastrointestinal tract were examined. Gastrointestinal segments (esophagus, stomach, small intestine, large intestine, and cecum) were opened and rinsed under running water. The gastrointestinal contents were sieved through a mesh sieve with a 0.075-mm aperture. All material retained on the sieve was immediately inspected under a stereomicroscope (Nikon SMZ745, Nikon, Japan) for helminth recovery. The collected specimens were preserved in 70% ethanol and separated according to organ, host, and locality.

During the necropsy of a bush dog (*S. venaticus*), parasites were found in the gastric cavity, along with multiple nodular lesions in the stomach wall. A fragment of nodular tissue (approximately 2 × 2 × 1 cm) was collected and submitted for histopathological examination. The sample was fixed in 10% buffered formalin and routinely processed with paraffin embedding, sectioned at 5 μm, and stained with hematoxylin and eosin (H&E) [18]. Slides were examined by brightfield microscopy (Nikon Eclipse SI, Nikon, Shinagawa, Japan) using 4× and 10× objective lenses.

For morphological identification, the parasites were rehydrated and cleared in 50% glycerol solution or lactophenol for up to 24 h, depending on specimen thickness. More robust helminths were cleared in 90% phenol solution, following the method of Hoffmann [19]. Acanthocephalans and trematodes were stained with carmine acid, followed by destaining in 0.5% hydrochloric acid (HCl) in 70% ethanol, dehydration through a graded ethanol series, and clearing with eugenol (4-allyl-2-methoxyphenol), following the modified protocol described by Amato [20].

Temporary slides were examined under a light microscope. Morphological structures relevant to taxonomic identification were documented using a Nikon Eclipse SI RS light microscope with an attached PrimeCam Intervision NPlus 12 camera. Helminth identification was based on taxonomic keys and descriptions specific to each parasitic group: Vicente et al. [21], Anderson et al. [22], and Gibbons [23] for nematodes; Khalil et al. [24] for cestodes; Travassos et al. [25] and Gibson et al. [26] for trematodes; and Schmidt [27] for acanthocephalans. This enabled classification to the most specific taxonomic level possible according to sample viability. Occurrence (Oc), mean intensity (MI) of parasitism, and mean abundance (MA) were calculated as described by Bush et al. [28].

## 3. Results

Of the 19 animals examined, 16 (84.2%) harbored at least one helminth species. A total of 1041 helminths were collected and identified, representing 17 taxa distributed across 11 genera. The mean abundance was 54.8 parasites per host. Individual specimen data and accession numbers deposited in the Helminthological Collection of UFJ are listed in Table 1.

### 3.1. Helminthofauna

#### 3.1.1. Nematoda

##### *Toxocara canis* (Werner, 1782) Stiles, 1905

Nematodes of the genus *Toxocara* are characterized by the absence of interlabia, an esophagus bearing a distinct posterior muscular ventriculus, delicate rows of denticles occasionally present, and evident sexual dimorphism. In males, subulate alate spicules are present, the gubernaculum is absent, and a terminal digitiform appendage is associated with numerous papillae (five post-anal papillae, one double subventral papilla between the cloaca and the base of the appendage, and approximately 20 pre-anal papillae on each side). Females have the vulva located in the anterior quarter of the body, with the vagina directed posteriorly, and a reproductive system composed of two uterine branches. Species are oviparous and produce subglobular to globular eggs with a rough outer surface.

**Figure 2:**
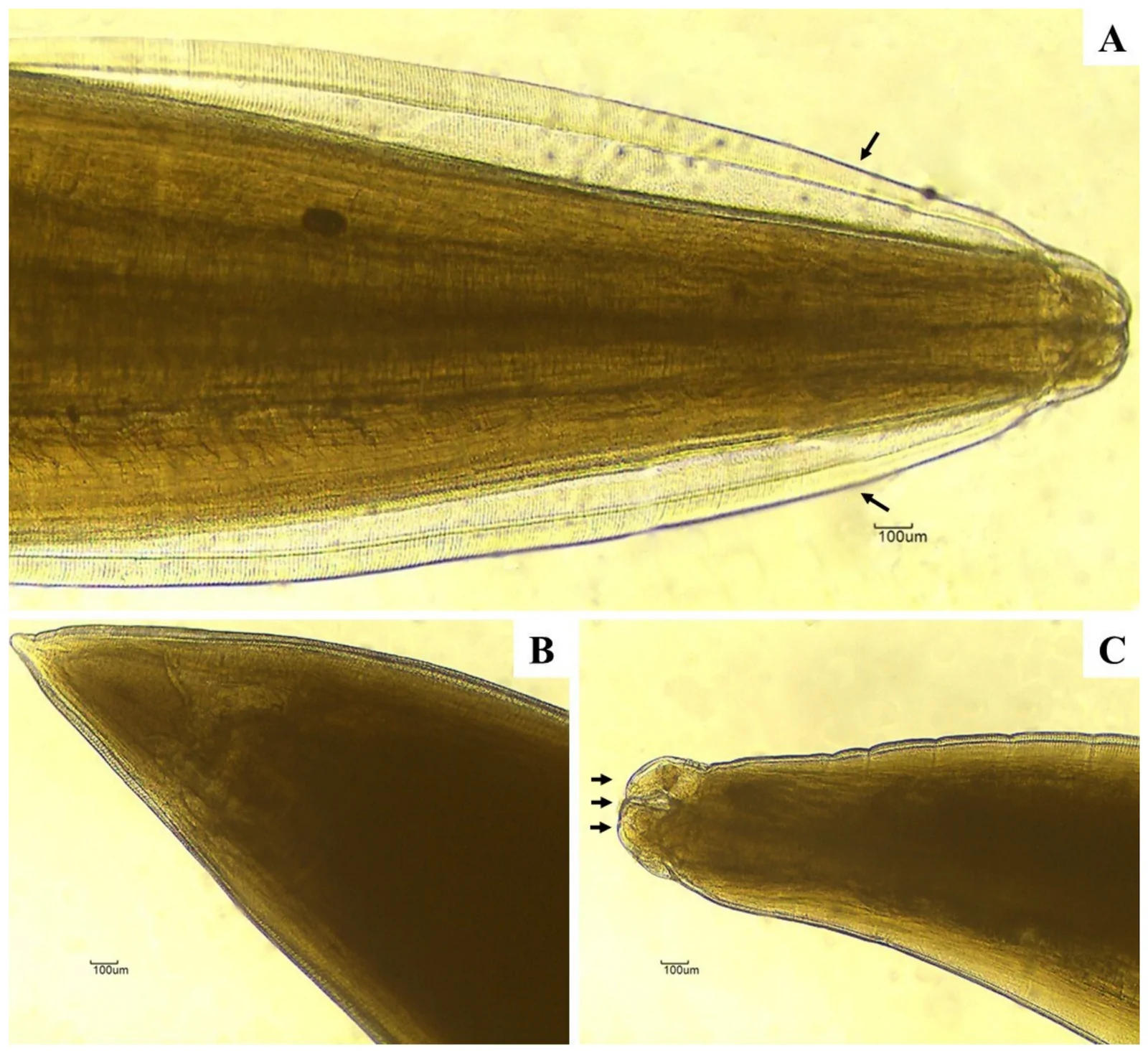
Micrograph of *Toxocara canis* from *C. thous*. (**A**) Cervical alae of *T. canis* (black arrows); (**B**) posterior view of a female, showing a straight, tapering tail; (**C**) anterior view of a female showing trilobate lips (black arrows). Scale bar = 100 µm.

Specimens were characterized by the presence of cervical alae, three well-developed lips with two distinct lateral lobes, and one unpaired internal lobe (Figure 2). The presence of a ventriculus located between the esophagus and the intestine was also noted—a structure absent in the genus *Toxascaris*. Males presented exteriorized, distally curved spicules and a digitiform process (Figure 2), whereas females had a straight, tapering tail without digitiform projections, demonstrating sexual dimorphism. The cervical alae of *T. canis* were narrower and tapered gradually compared with those of other species in this genus (Figure 2).

Hosts: *S. venaticus*; *C. thous*.

Locality: Vila Bela da Santíssima Trindade, MT (Amazonian transition zone); Nova Mutum, MT (Cerrado); Poconé, MT (Pantanal).

##### *Ancylostoma* Dubine, 1843

Nematodes of the genus *Ancylostoma* are characterized by a deep buccal capsule bearing ventral teeth and triangular lancets in its interior. Sexual dimorphism is evident. Males possess a well-developed copulatory bursa with a small dorsal lobe, a characteristic pattern of bursal ray distribution, equal spicules, and a present gubernaculum. Females have the vulva located in the posterior half of the body and a reproductive system composed of opposing uterine branches.

**Figure 3:**
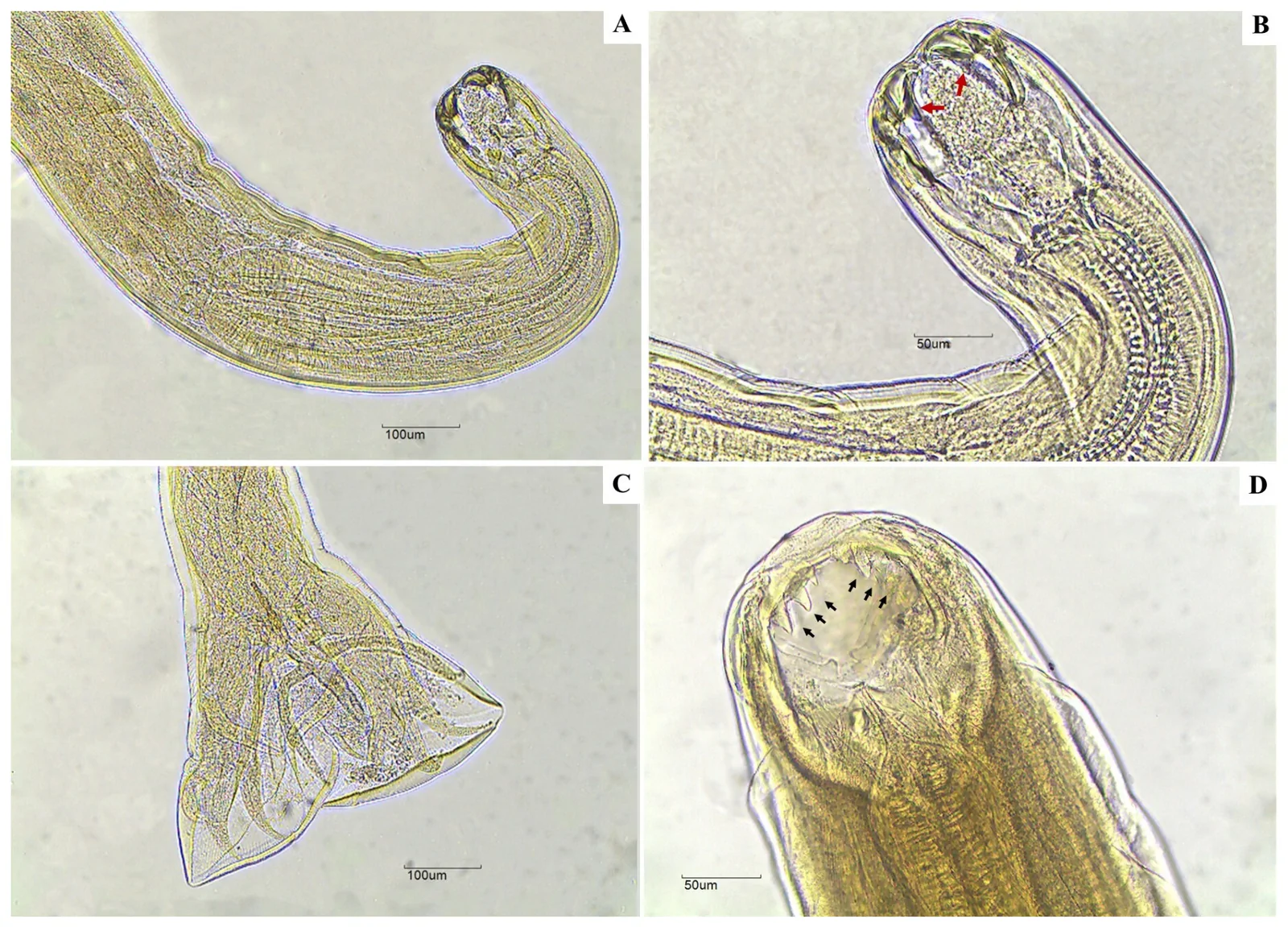
Micrograph of ancylostomatid nematodes recovered from wild canids. (**A**) Anterior view of *Ancylostoma braziliense* parasitizing *Cerdocyon thous*. Scale bars = 100 µm. (**B**) Larger teeth of the buccal capsule (red arrows). Scale bars = 50 µm. (**C**) Posterior view, male of *A. braziliense*. Scale bar = 100 µm. (**D**) Anterior view of *Ancylostoma caninum* parasitizing *Chrysocyon brachyurus* with three pairs of ventral teeth (black arrows). Scale bar = 50 µm.

Specimens exhibited a dorsally curved anterior extremity, a well-developed buccal capsule with teeth, plates, lancets, or cutting laminae, and a muscular esophagus. Males had a well-developed copulatory bursa (Figure 3). Species-level identification was precluded by morphological degradation.

Host: *C. thous*.

Locality: Vila Bela da Santíssima Trindade, MT (Amazônia); Cuiabá, MT (Cerrado).

*Ancylostoma caninum* (Ercolani, 1859) Hall, 1913.

Specimens were rigid-bodied with a dorsally curved anterior extremity and a deep buccal capsule bearing three pairs of teeth along the ventral margin and one pair of triangularly shaped dorsal teeth (Figure 3). Identification was confirmed by the presence of centrolateral teeth, which distinguishes this species from other congeners.

Host: *C. brachyurus*.

Locality: Poconé, MT (Pantanal); Mineiros, GO (Cerrado).

##### *Ancylostoma braziliense* Faria, 1910

This species was distinguished from other species within the same genus by the presence of two ventral dental plates, each bearing a single tooth (Figure 3).

Host: *C. thous*.

Locality: Jataí-GO (Cerrado); Cuiabá-MT (Cerrado).

##### *Spirocerca lupi* (Rudolphi, 1809) Railliet & Henry, 1911

The genus *Spirocerca* exhibits an esophagus divided into a narrow anterior muscular portion and a more developed posterior glandular portion, cervical papillae situated near the nerve ring, and a well-developed reproductive system. Species are oviparous and display evident sexual dimorphism, with males and females readily distinguishable by the conformation of the caudal extremity (coiled in males and rounded in females) and the reproductive system (presence of unequal spicules in males).

**Figure 4:**
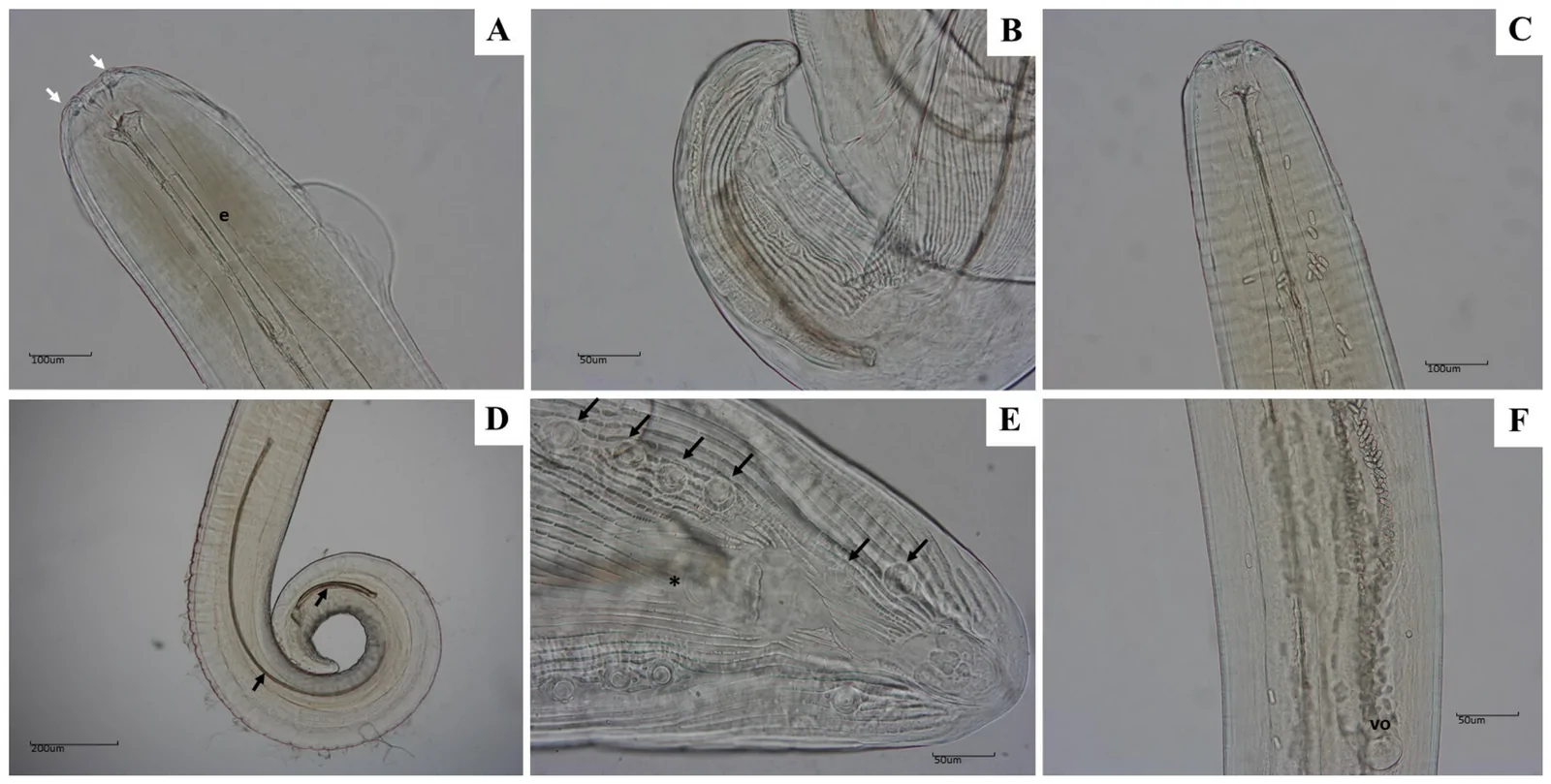
Micrographs of *Spirocerca lupi* in *Speothos venaticus*. (**A**) Anterior portion of a male, showing the cephalic papillae (white arrows) and esophagus (e). Scale bar = 100 µm. (**B**,**E**) Sessile papillae in the posterior portion of a male (black arrows), and a single pre-cloacal papilla on the anterior lip of the cloacal opening (asterisk). Scale bar = 50 µm. (**C**) Anterior portion of an egg-laden female. Scale bar = 100 µm. (**D**) Caudal portion of a male, showing the spicules (black arrows). Scale bar = 200 µm. (**F**) Median portion of an egg-laden female, showing the vulvar opening (vo). Scale bar = 50 µm.

Specimens of *S. lupi* exhibited a hexagonal buccal capsule with thick, smooth walls, lacking rugosities, dentition, or defined lips, two pairs of submedian lateral cephalic papillae, and one pair of lateral amphids (Figure 4). Males had a coiled tail with well-developed caudal alae and two spicules of unequal length (the longer being approximately four times the length of the shorter) (Figure 4), four pairs of pedunculate pre-anal papillae, one broad unpaired papilla anterior to the anus, two pairs of post-anal papillae, and four pairs of small papillae near the terminal caudal extremity (Figure 4).

Females exhibited a vulva obscured by the egg-filled uterus. The uterus extended to the anterior region of the esophagus and then folded ventrally, forming folds that resembled the vulval opening. Eggs were cylindrical with a thick shell, containing embryos at the time of expulsion (Figure 4). Adults parasitized nodular cysts in the esophagus, stomach, aorta, and lungs of carnivores and marsupials.

Host: *S. venaticus*.

Locality: Jataí, GO (Cerrado).

##### *Physaloptera* Rudolphi, 1819

Representatives of this genus possess a short or absent buccal cavity, two large simple triangular lateral lips, an esophagus divided into an anterior muscular and a posterior glandular portion, and marked sexual dimorphism. Females have two or more parallel uteri, with the vulva located anterior or posterior to the midbody, and are oviparous, producing thick-shelled eggs that are embryonated at oviposition. Males bear wide caudal alae, papillae associated with copulatory structures (three to seven pairs of large papillae in the cloacal region and a variable number of sessile ventral papillae around the cloacal opening and on the tail), and equal or unequal spicules.

Specimens of this genus were identified by large triangular lateral lips armed with one or more teeth at the anterior extremity, a cuticle folded over the lips forming a cephalic collarette, and a bipartite esophagus. Males had well-developed caudal alae, united ventrally anterior to the cloaca and supported by elongated pedunculate papillae, evidencing sexual dimorphism. Species-level identification was precluded by morphological degradation.

Host: *C. thous*.

Locality: Jataí, GO (Cerrado).

##### *Physaloptera digitata* Schneider, 1866

**Figure 5:**
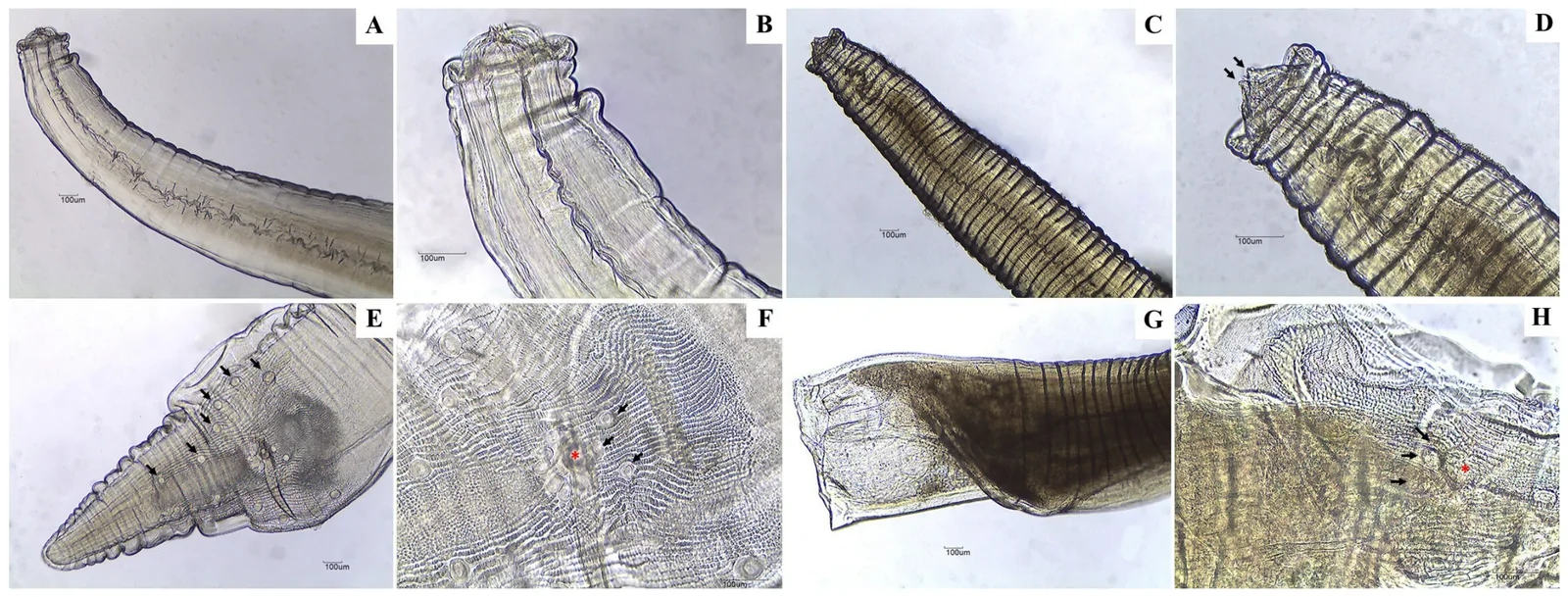
Micrograph of *Physaloptera* spp. recovered from *Cerdocyon thous*. (**A**,**B**) Anterior portion of *Physaloptera digitata*; (**C**,**D**) anterior portion of *Physaloptera praeputialis*, two large, simple, triangular lateral lips (black arrow); (**E**) posterior portion of a male *P. digitata* showing papillae (black arrow); (**F**) sessile precloacal papillae in a triangular arrangement (black arrow) and cloaca (asterisk); (**G**) posterior portion of a male showing well-developed caudal alae of *P. praeputialis*; (**H**) posterior portion of a male *Physaloptera terdentata* showing sessile precloacal papillae (black arrows) and cloaca (asterisk). Scale bar = 100 µm.

Specimens of this species exhibited a cuticularized buccal cavity and a cuticular dilation in the posterior body region with folds. Males presented well-developed pedunculate papillae arranged in three pairs of pre-cloacal and one pair of post-cloacal papillae, in addition to sessile papillae distributed as three pre-cloacal papillae in a triangular pattern, four papillae below the cloaca, and three pairs distributed in the posterior caudal region (Figure 5).

Host: *C. thous*.

Locality: Jataí, GO (Cerrado).

##### *Physaloptera praeputialis* Linstow, 1889

Specimens of this species exhibited a body with a rugose cuticle and transverse striations. Anterior triangular lips with small teeth and a pleated cuticular sheath projecting anteriorly as a prepuce-like cephalic collarette were identified (Figure 5). Males had well-developed caudal alae (Figure 5) and sessile papillae distributed as three pre-cloacal, six post-cloacal, two adjacent to the cloaca, and two at the posterior extremity.

Host: *C. thous*.

Locality: Jataí, GO (Cerrado); Poconé, MT (Pantanal).

##### *Physaloptera terdentata* Molin, 1860

Specimens were differentiated by males with a body tapering in the anterior portion, cuticle slightly reflected over the lips, fine transverse striations on the outer surface, and an elongated copulatory bursa with rounded extremities bearing four pairs of pedunculate papillae (two pre-cloacal and two post-cloacal) (Figure 5), seven sessile papillae around the cloaca (three pre-cloacal and four post-cloacal), and three pairs of papillae at the terminal portion of the tail.

Host: *C. thous*.

Locality: Jataí, GO (Cerrado).

##### *Dioctophyme renale* (Goeze, 1782)

The genus *Dioctophyme* Collet-Meygret, 1802 is represented exclusively by *D. renale*. Adults have a cylindrical, intensely red body covered by a thin cuticle, with marked sexual dimorphism: females measure 100–103 cm in length and 6–12 mm in diameter; males are approximately 35 cm in length and 3–5 mm in width. The anterior extremity bears a hexagonal oral aperture. In females, the tail is narrow and abruptly truncated, with the vulva located posteriorly, near the esophageal extremity.

Host: *C.brachyurus*.

Locality: Jataí, GO (Cerrado).

##### *Pterygodermatites* Wedl, 1861

Nematodes of this genus bear cuticular spines arranged in subventral rows along the body, or at least in the anterior region, becoming sparser and smaller posteriorly. The oral aperture is in an apical or dorsally displaced position, and three esophageal teeth are present. Marked sexual dimorphism is evident; the vulva is located in the anterior region of the body in females. Males are characterized by the presence of caudal papillae and copulatory structures.

Specimens of *Pterygodermatites* spp. were characterized by a cuticle armed with chitinous spines in rows, three esophageal teeth, and 29–58 pairs of pre-vulvar spines. Species-level identification was not possible owing to specimen deterioration.

Hosts: *C. thous*; *C. brachyurus*.

Locality: Jataí, GO; Mineiros, GO (Cerrado).

##### *Pterygodermatites (Multipectines) affinis* Jägerskiöld, 1904

**Figure 6:**
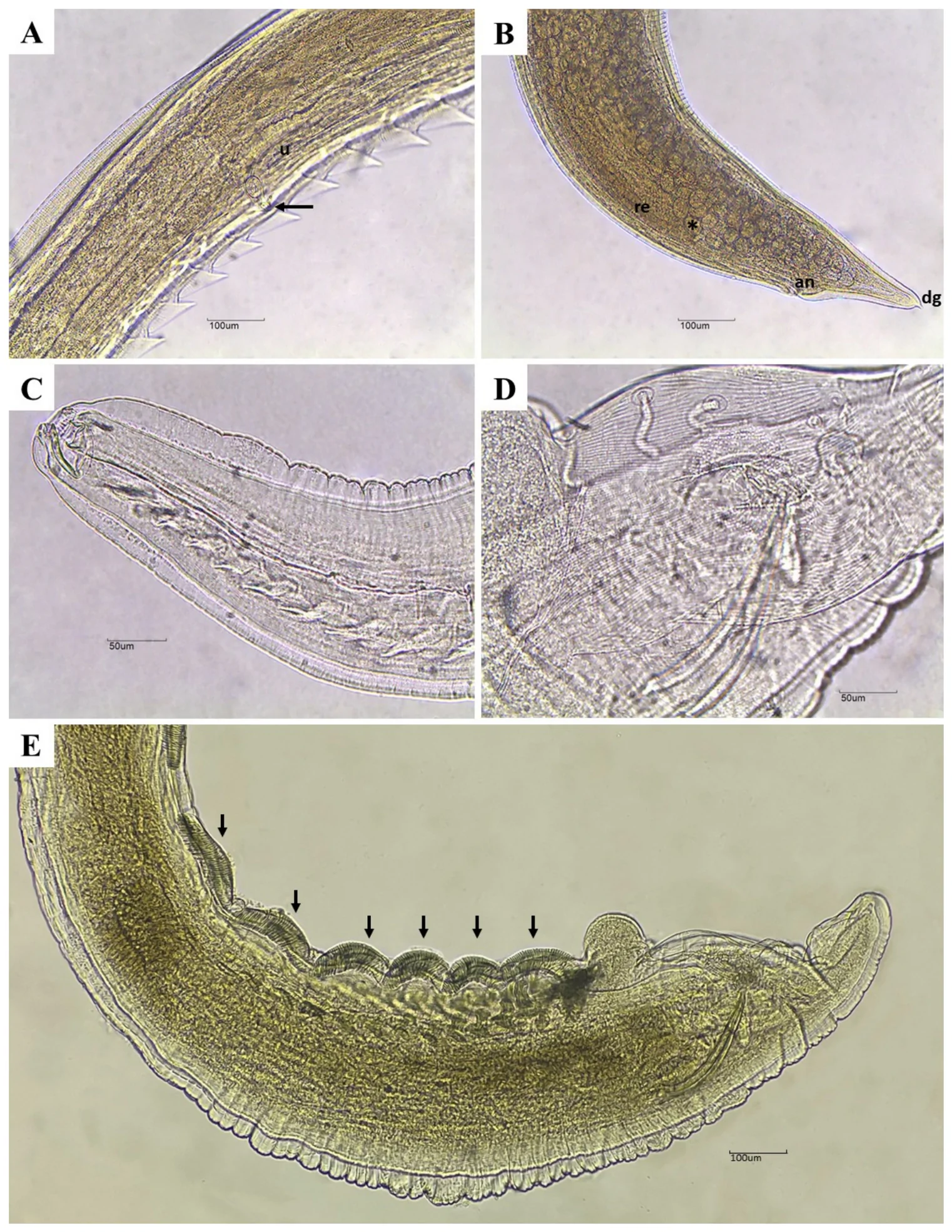
Micrograph of *Pterygodermatites* (*M.*) *affinis*. (**A**) Vulval opening (arrow), followed by the uterus (u). (**B**) Posterior extremity of a female in lateral view: eggs (*), rectum (re), anal opening (an), digitiform cuticular projection (dg). (**C**) Overview of the anterior portion in ventral view: open buccal capsule with denticles. (**D**) Enlarged lateral view of the posterior extremity of a male, highlighting papillae. (**E**) Overview of the posterior region of a male: cloacal opening, spicules, gubernaculum present, caudal alae and ventral cuticular fans (arrows). Scale bar = 100 µm.

Specimens of this species were medium-sized nematodes with an apical oral aperture and three well-developed esophageal teeth (Figure 6). Males had 102–114 pairs of subventral cuticular projections, a coiled posterior extremity with a ventral groove, and two delicate lateral alae supported by three pairs of pedunculate pre-cloacal papillae and seven pairs of post-cloacal papillae (six pedunculate and one sessile at the tail tip) (Figure 6). Two of the six pairs of pedunculate post-cloacal papillae were positioned more medially, adjacent to the ventral groove. The cloacal aperture was surrounded by rounded verruciform projections and bore a large sessile papilla (Figure 6). The spicules were slender and short, and the gubernaculum was well sclerotized with a spoon-shaped profile in lateral view. Females exhibited two rows of pre-vulvar spines and a uterus containing eggs, indicating reproductive activity (Figure 6).

Hosts: *C. thous*, *L. vetulus*.

Locality: Jataí, GO (Cerrado).

#### 3.1.2. Cestoda

##### *Spirometra* Manson, 1882 (Plerocercoid)

**Figure 7:**
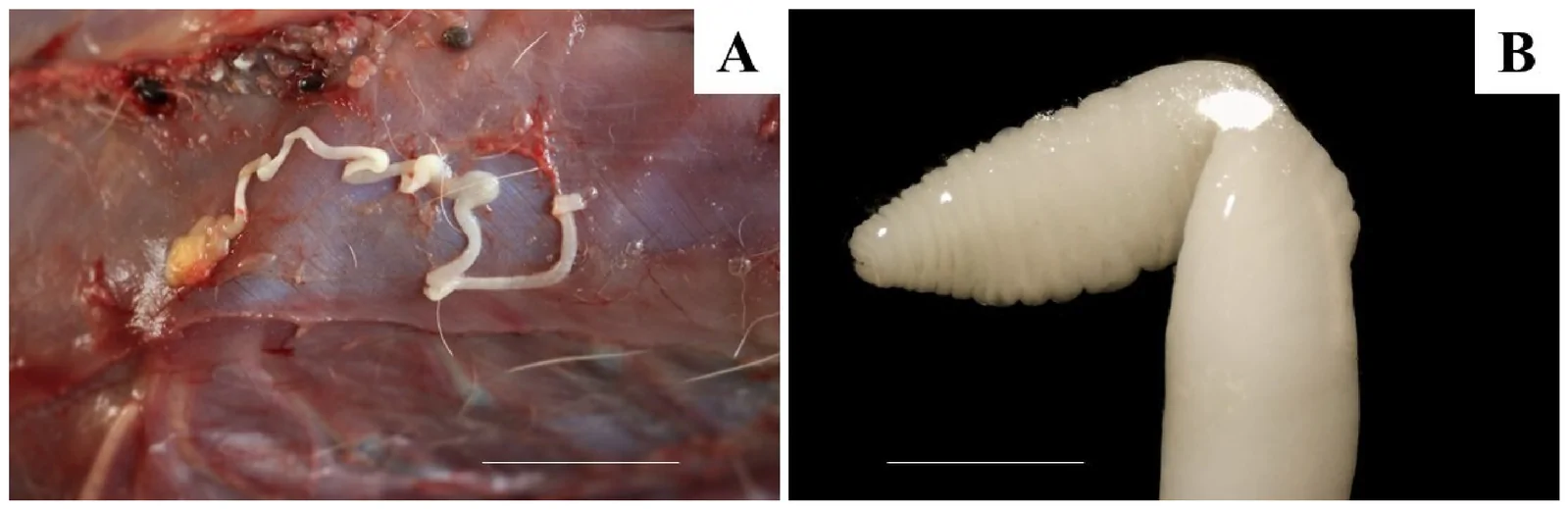
Gross and microscopic findings associated with *Spirometra* sp. infection in *Cerdocyon thous*. (**A**) Gross appearance of the plerocercoid recovered from the intercostal musculature, showing its elongated, coiled, whitish body. Scale bar = 2 cm. (**B**) Higher magnification of the frontal segment of *Spirometra* sp. under the stereomicroscope. Scale bar = 2 mm.

This genus is characterized by the presence of a cirrus sac associated with a seminal vesicle and plerocercoid larvae (spargana), which are infective to all vertebrate groups except fish. The spargana were white, flattened, ribbon-like larvae measuring up to 50 cm in length, with wider regions alternating with narrow, filiform portions, a solid internal structure, a lack of a scolex, differentiated internal organs, and a strobila (Figure 7).

Host: *C. thous*.

Locality: Poconé, MT (Pantanal).

#### 3.1.3. Trematoda

##### *Athesmia foxi* Goldberger & Crane, 1911

Representatives of this genus are characterized by oral and ventral suckers of similar size, located in the anterior quarter of the body, a relatively long esophagus, and ceca extending to the posterior half of the body without reaching the caudal extremity. The male reproductive system comprises testes arranged in tandem in the anterior region of the body and a small, elongated cirrus sac. The female reproductive system includes a submedian ovary, a tightly coiled uterus posterior to the ovary, and a vitellarium consisting of a single band of follicles distributed posterior to the ovary. The excretory vesicle is I- or Y-shaped, and the stem may reach approximately the level of the anterior extension of the vitellarium.

**Figure 8:**
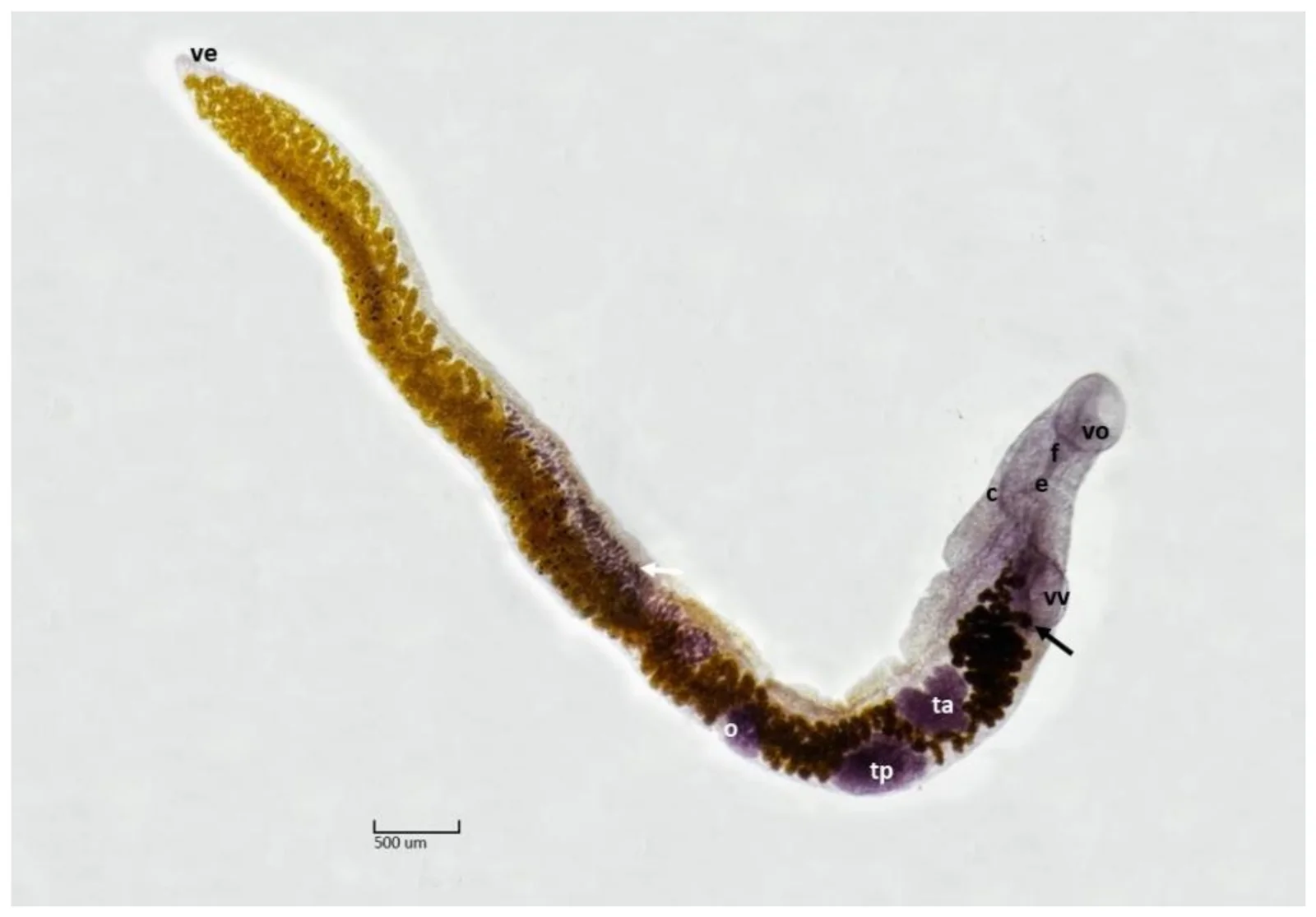
Micrograph of *Athesmia foxi* from *Lycalopex vetulus*. (vo) oral sucker; (f) pharynx; (e) esophagus; (c) cecum; (vv) ventral sucker; (black arrow) uterus; (ta) anterior testis; (tp) posterior testis; (o) ovary; (white arrow) vitellarium; (ve) excretory vesicle. Scale bar = 500 µm.

Specimens were identified by a relatively large, slender, aspinose, and elongated body (Figure 8), with a rounded anterior extremity conforming to the shape of the oral sucker and a pointed posterior extremity. The body was visibly narrower anterior to the genital pore, and this anterior portion was slightly wider than the oral sucker. The uterus was confined between the ceca or extended laterally to one or both diverticula.

Hosts: *L. vetulus*, *C. thous*, *C. brachyurus*.

Locality: Jataí, GO (Cerrado).

#### 3.1.4. Acanthocephala

##### *Oncicola* sp. Travassos, 1916

Representatives of this genus are characterized by a trunk that is relatively wide in proportion to its length, with the anterior trunk slightly wider than the posterior trunk. The proboscis is subspheroidal, bearing 12 longitudinal rows, regularly alternating and approximately straight, each with six hooks. Proboscis hooks possess well-developed manubria and roots. In males, the cement glands are compact and generally composed of eight units, each containing a giant nucleus. Females produce eggs with an ornate outer shell, a feature frequently employed in group identification.

**Figure 9:**
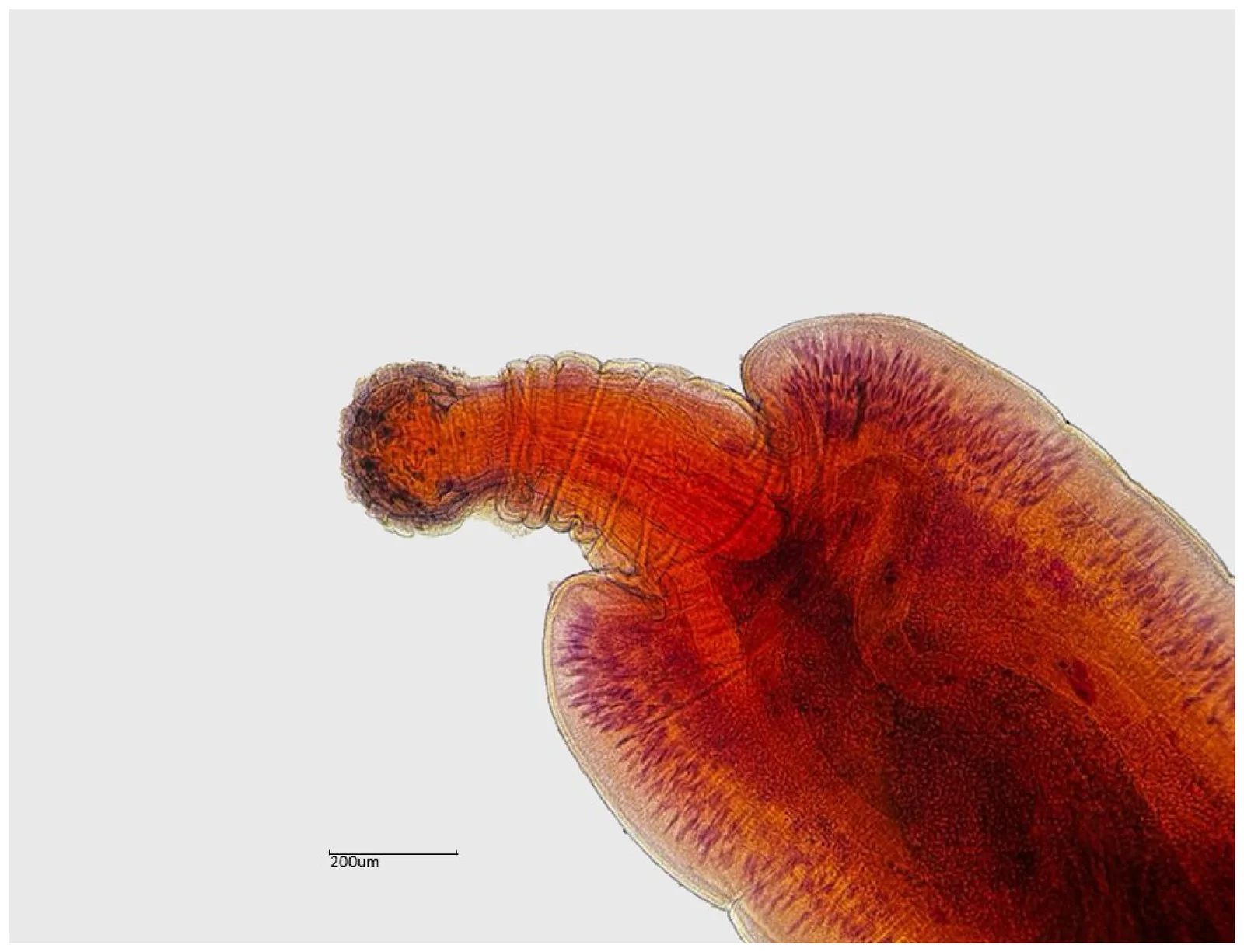
Micrograph of the anterior portion of a male *Oncicola* spp., proboscis with hooks. Scale bar = 200 µm.

Specimens of this genus are identified by a trunk with a globose and cylindrical anterior portion that gradually elongates toward the posterior extremity and a retractile proboscis bearing six longitudinal rows of six hooks each (Figure 9). The absence of a cervical collar distinguishes them from the genus *Prosthenorchis*. The proboscis was anchored in a single-walled receptacle connected to the body by a short neck. Males possessed a copulatory bursa, two testes arranged in anterior and posterior positions, and cement glands. Females exhibited a well-developed uterus extending into a bell-shaped structure connected to the vagina and vulva.

Hosts: *C. thous*; *S. venaticus*.

Locality: Nova Mutum, MT; Jataí, GO (Cerrado).

##### Oligacanthorhynchidae Petrochencko, 1956

**Figure 10:**
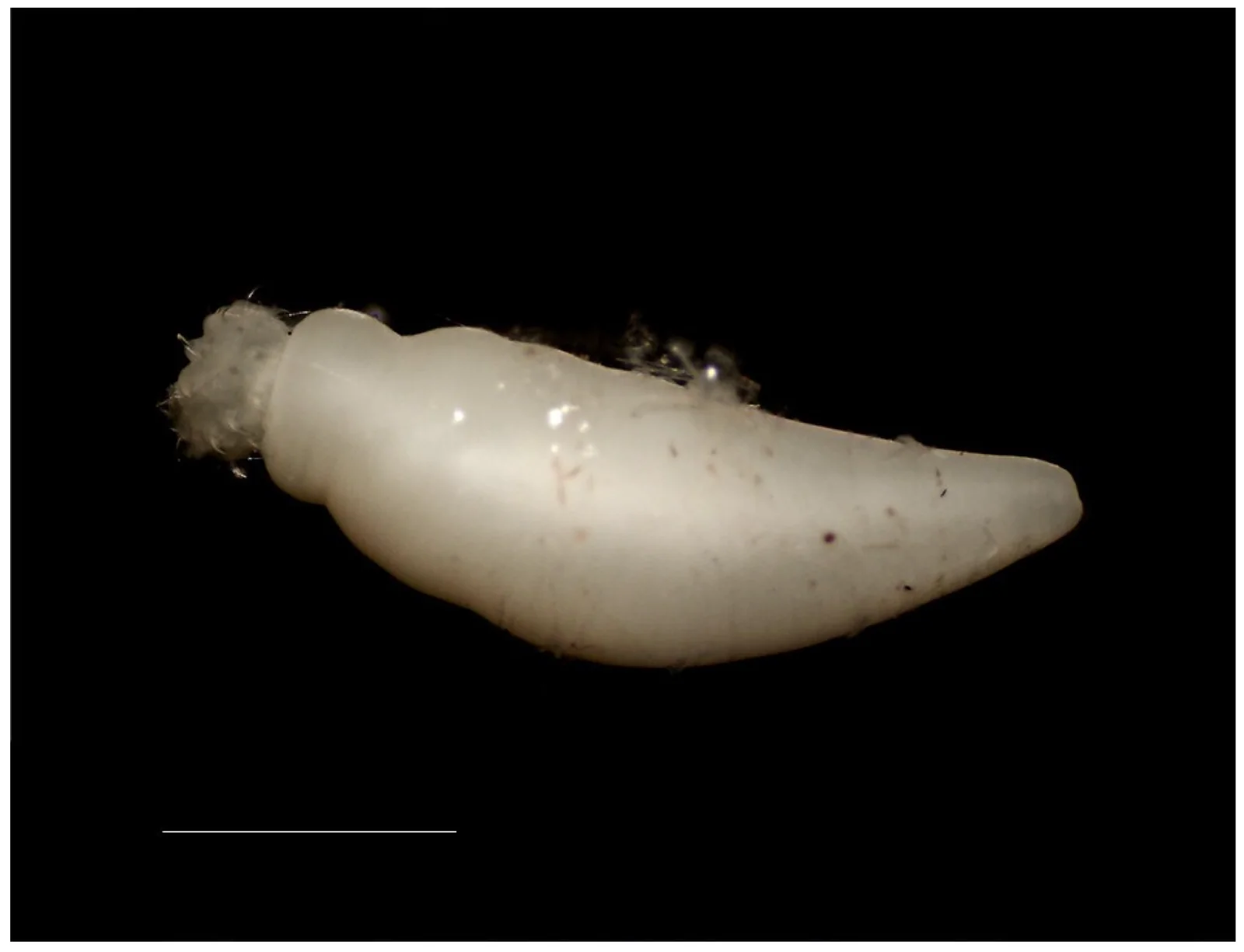
Specimen of the family Oligacanthorhynchidae, globular proboscis bearing hooks, observed under a stereoscopic light microscope. Scale bar = 200 µm.

Specimens collected from *C. thous* were assigned to the class Archiacanthocephala based on a globular proboscis bearing a reduced number of hooks arranged spirally in oblique rows and the absence of trunk spines (Figure 10). Assignment to the family Oligacanthorhynchidae was based on the few-hooked proboscis and a short receptacle not embedded in the anterior region of the body.

Host: *C. thous*.

Locality: Cuiabá, MT (Cerrado).

##### *Sphaerisrostris* sp. Golvan, 1956

**Figure 11:**
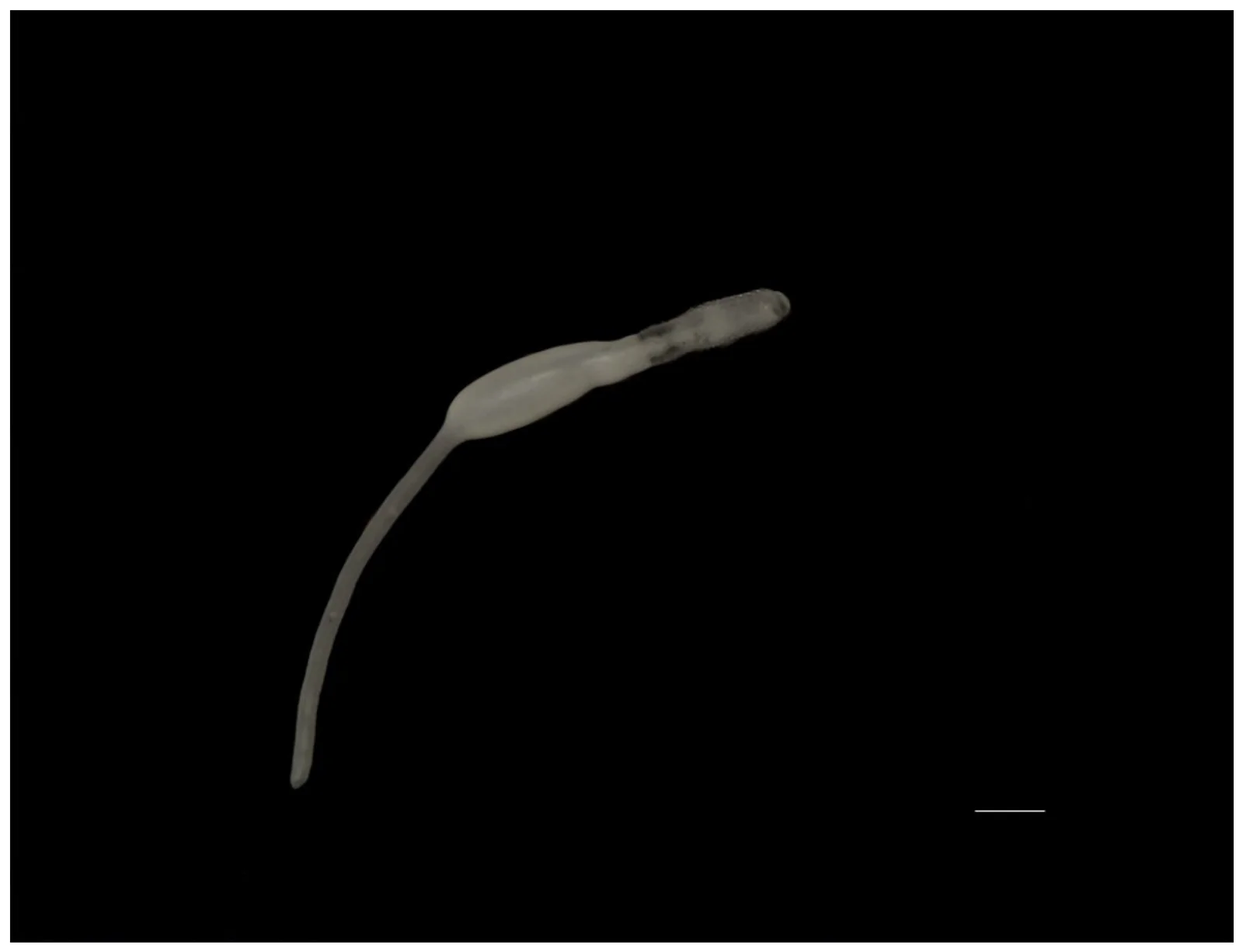
*Sphaerirostris* sp. observed under a stereoscopic light microscope. Scale bar = 40 µm.

The description is based on six specimens. The body is elongated and filiform. The proboscis is divided into three portions, the first quadrangular, the second inflated, the third elongated and wider, with a constriction at the insertion of the double-walled proboscis receptacle. The proboscis has 28–30 longitudinal rows of hooks, each row comprising 21–23 hooks (Figure 11).

Host: *C. thous*.

Locality: Cuiabá, MT (Cerrado).

### 3.2. Histopathological Lesions Caused by Spirocerca lupi in Speothos venaticus

*Spirocerca lupi* is a parasite primarily affecting dogs, although other carnivores may also be infected [29]. The adult parasite is typically found within a nodular mass in the wall of the thoracic esophagus of the host. *S. lupi* has an indirect life cycle, in which carnivores become infected by ingesting intermediate hosts (coprophagous beetles) or paratenic hosts (birds, hedgehogs, lizards, mice, and rabbits) [30]. Larvae migrate through the gastric wall and the thoracic aorta to the caudal esophagus after ingestion, where they develop into adult worms and induce the formation of esophageal nodules. This may progress from fibrous and inflammatory lesions to neoplastic lesions of the esophagus [31]. Although *S. lupi* has been reported in domestic dogs in the Neotropical region, there are no reports of its occurrence causing gastric nodules in exotic animals. This study documents the first case of *S. lupi* inducing gastric nodular formations in *S. venaticus* in South America.

**Figure 12:**
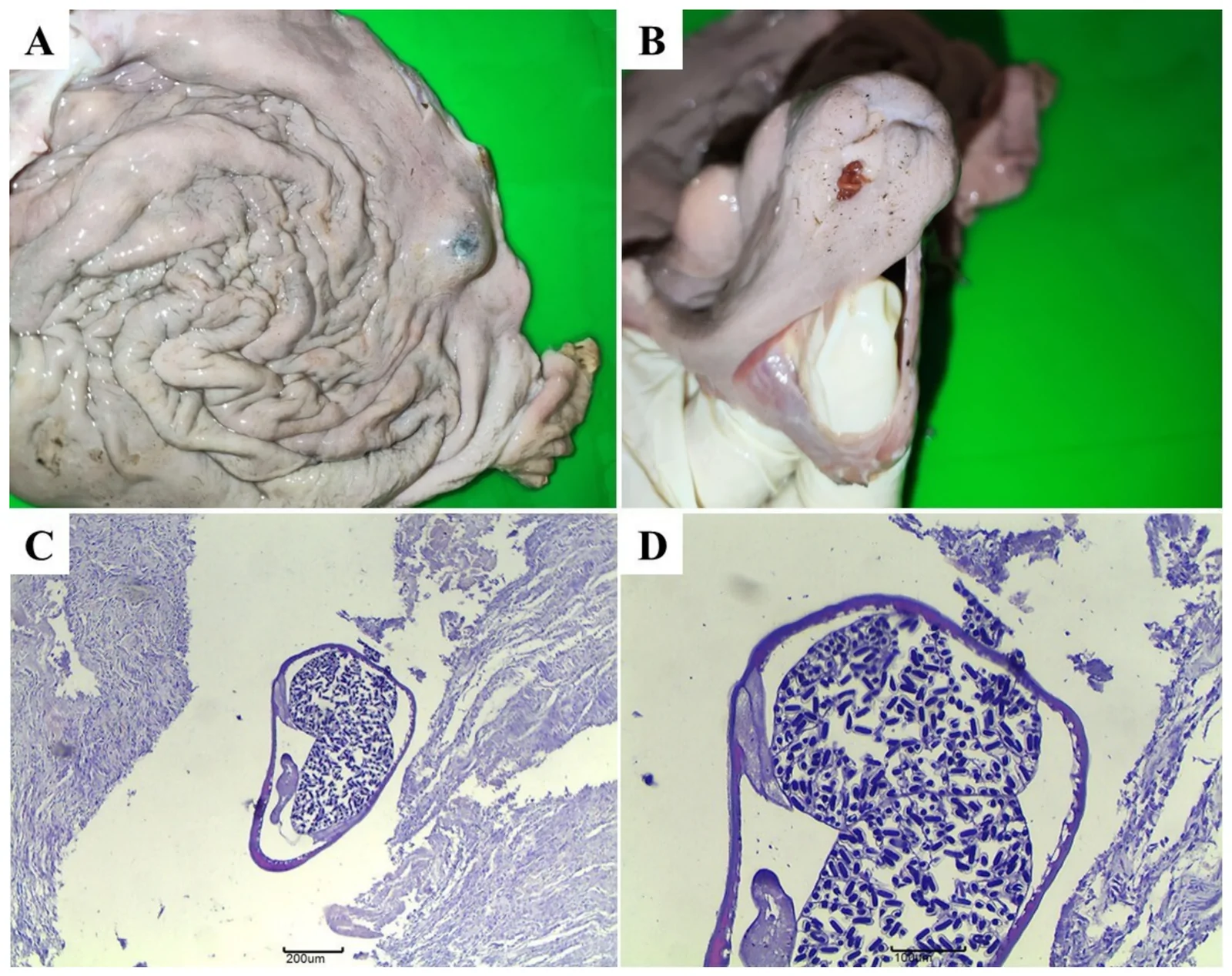
Gross and microscopic lesions associated with *Spirocerca lupi* infection in *Speothos venaticus*. (**A**) Gross pathology showing a nodule in the gastric wall (arrow). (**B**) Gastric nodule with a central ulcerated area and the presence of *S. lupi* specimens. (**C**) Muscular wall of the stomach with marked fibrosis and moderate inflammatory infiltrate composed of neutrophils. H&E, 4× objective, scale bar: 200 µm. (**D**) Cross-section of a parasitic structure, measuring approximately 600 µm along its greatest axis, showing the external cuticle and reproductive system containing eggs. H&E, 10× objective, scale bar = 100 µm.

Macroscopically, the gastric sample consisted of a nodular mass approximately 3 cm in diameter along its longest axis, grayish in color with a blackened central area (Figure 12). It appeared solid on sectioning, with parasites present. Histologically, the gastric musculature exhibited fibrosis with a moderate inflammatory infiltrate. Cross-sections of the parasite, approximately 600 micrometres in diameter at their largest axis, were observed within the lesion. These displayed an external cuticle and a visible reproductive system containing ellipsoid, embryonated eggs (Figure 12). The cause of death was trauma; the gastric parasites were an incidental finding.

Most *S. lupi* parasitic nodules are located in the caudal esophagus and contain coiled adult worms within. However, aberrant migration may occur to other sites, including the stomach, intestine, mediastinum, kidneys, and urinary bladder. Only 3–5% of infected domestic dogs presented an intramural gastric nodule at necropsy [32].

### 3.3. General Parasitism

Occurrence (Oc) with confidence interval (CI), mean intensity (MI), mean abundance (MA) and total count (TC) were calculated. Overall, 16 of the 19 animals (Oc 84.2%; 95% CI: 60.4–96.6) were infected with at least one helminth species. A total of 1041 helminths were recovered, with an MA of 54.8 parasites (Table 2). The following helminth species were identified: *T. canis* (Oc 15.8%; 95% CI 3.4–39.6; TC 42; MI 14; MA 2.2), *A. braziliense* (Oc 10.5%; 95% CI 1.3–33.1; TC 10; MI 5; MA 0.5), *A. caninum* (Oc 10.5%; 95% CI 1.3–33.1; TC 619; MI 309.5; MA 32.6), *Ancylostoma* spp. (Oc 10.5%; 95% CI 1.3–33.1; TC 3; MI 1.5; MA 0.2), *S. lupi* (Oc 5.3%; 95% CI 0.1–26.0; TC 13; MI 13; MA 0.7), *P. digitata* (Oc 10.5%; 95% CI 1.3–33.1; TC 23; MI 11.5; MA 1.2), *P. praeputialis* (Oc 15.8%; 95% CI 3.4–39.6; TC 23; MI 7.7; MA 1.2), *P. terdentata* (Oc 5.3%; 95% CI 0.1–26.0; TC 6; MI 6; MA 0.3), *Physaloptera* spp. (Oc 5.3%; 95% CI 0.1–26.0; TC 1; MI 1; MA 0.1), *P. affinis* (Oc 26.3%; 95% CI 9.2–51.2; TC 68; MI 13.6; MA 3.6), *Pterygodermatites* spp. (Oc 15.8%; 95% CI 3.4–39.6; TC 22; MI 7.3; MA 1.2), *D. renale* (Oc 15.8%; 95% CI 3.4–39.6; TC 9; MI 3; MA 0.5), *Spirometra* spp. (Oc 5.3%; 95% CI 0.1–26.0; TC 8; MI 8; MA 0.4), *A. foxi* (Oc 15.8%; 95% CI 3.4–39.6; TC 32; MI 10.7; MA 1.7), *Oncicola* spp. (Oc 21.1%; 95% CI 6.1–45.6; TC 158; MI 39.5; MA 8.3), Oligacanthorhynchidae (Oc 5.3%; 95% CI 0.1–26.0; TC 3; MI 3; MA 0.2), *Sphaerirostris* spp. (Oc 5.3%; 95% CI 0.1–26.0; TC 1; MI 1; MA 0.1).

**Figure 13:**
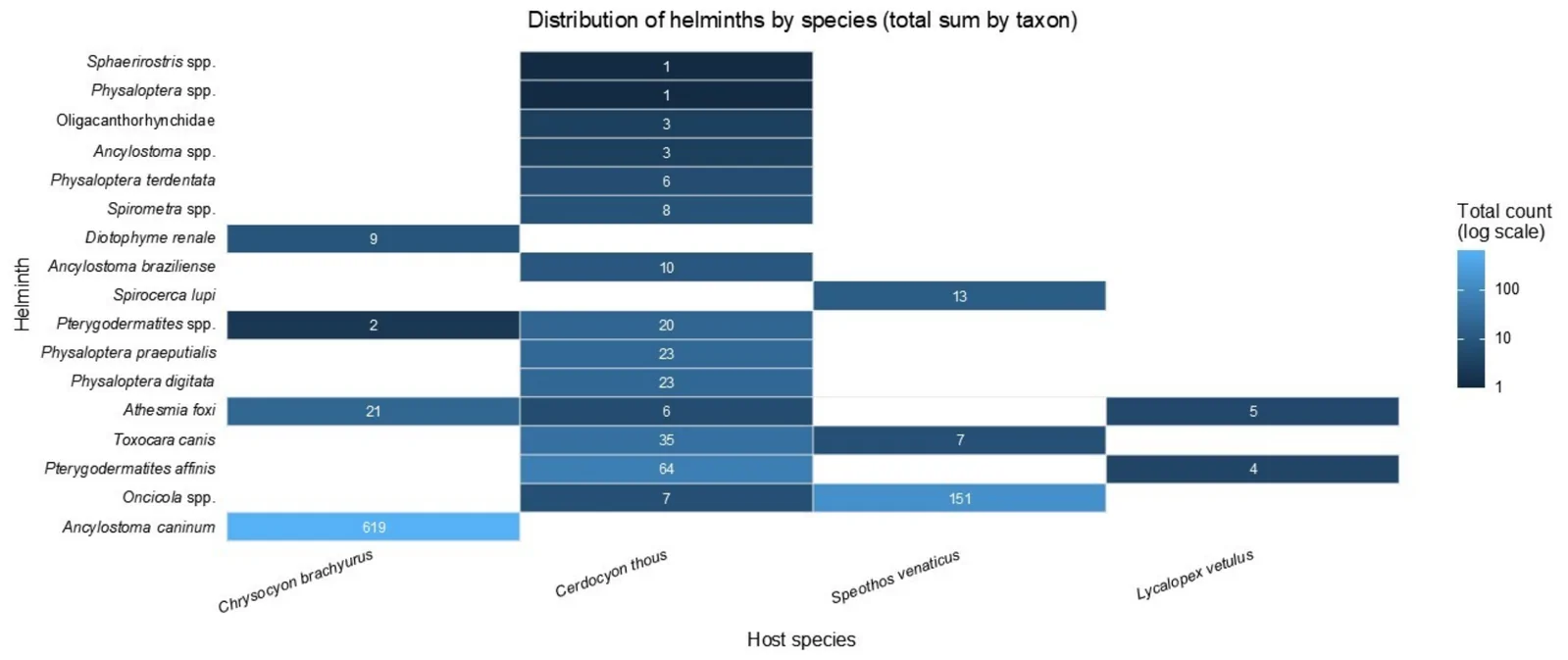
Heat map of the distribution and abundance of gastrointestinal helminths by host species (*Chrysocyon brachyurus*, *Cerdocyon thous*, *Speothos venaticus*, and *Lycalopex vetulus*). Color intensity represents the total number of specimens per taxon (logarithmic scale). Adapted using R Development Core Team (2013).

The three most abundant taxa were *A. caninum* (*n* = 619; MA 123.8) in *C. brachyurus*, *Oncicola* spp. (*n* = 151; MA 75.5) in *S. venaticus*, and *P. affinis* (*n* = 64) in *C. thous* (Figure 13). The helminth community exhibited an aggregated distribution pattern, in which most of the total parasite burden was concentrated in a small proportion of hosts. This was evidenced by *A. caninum* in *C. brachyurus*, where two of the five hosts were infected but harbored a mean of 309.5 parasites each.

The species *C. thous* exhibited the greatest helminth richness (14 taxa) and included representatives of all four identified classes: Nematoda, Cestoda, Trematoda, and Acanthocephala. *C. brachyurus* presented a high parasite burden of *A. caninum* (Oc 40%; MI 309.5; MA 123.8) and the presence of *D. renale* (Oc 60%; MI 3.0; MA 1.8). *S. venaticus* harbored *Oncicola* spp. in all examined individuals (Oc 100%; MI = MA = 75.5). Additionally, one individual of this species also presented *S. lupi* (Oc 50%; MI 13; MA 6.5) in the stomach, representing, to the best of our knowledge, the first record of this parasite in this host species in the Brazilian Cerrado. *L. vetulus* exhibited the lowest species richness, with only two taxa, i.e., *P. affinis* (Oc 50%; MI 4.0; MA 2.0) and *A. foxi* (Oc 50%; MI 5.0; MA 2.5), both representing new host records for this canid species. Plerocercoids of *Spirometra* spp. (sparganum) were recorded in one individual of *C. thous* (Oc 10.0%; MI 8; MA 0.8), representing the first report of sparganosis in this host species.

Helminth species richness varied among biomes, with the Cerrado presenting the greatest number of taxa (*n* = 16), followed by the Pantanal (*n* = 4) and the Cerrado–Amazon transition zone (*n* = 2) (Table 1). Statistical comparisons of richness among biomes were not performed owing to unequal host distributions across collection localities. Given the small sample sizes, no significant differences in occurrences were detected among host species for any taxon (Fisher’s exact test, all *p* > 0.05). No significant differences in infection intensity among host species were observed either (Mann–Whitney U test, all *p* > 0.05).

**Figure 14:**
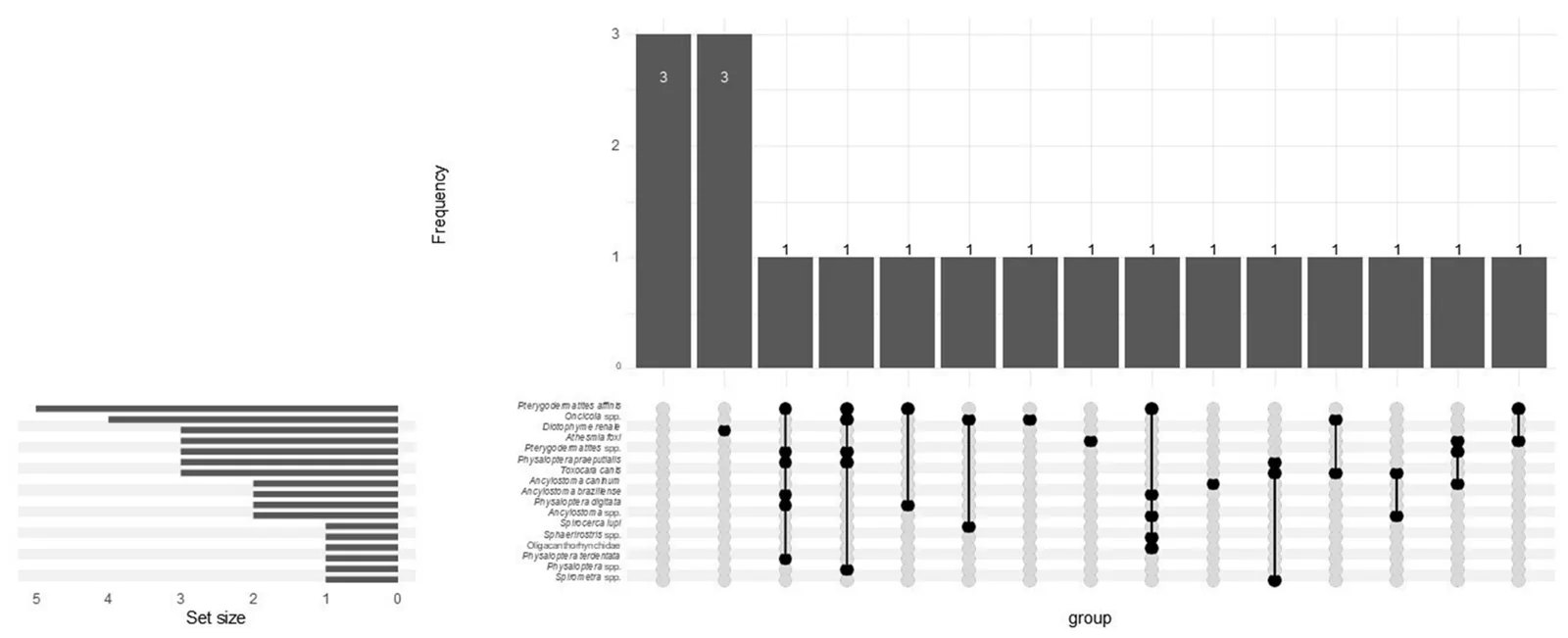
Co-infection patterns of helminths in wild canids, represented as an intersection plot (UpSet plot). The upper bars indicate the frequency of each combination of parasitic taxa, whereas the dot matrix identifies the sets of species involved in each infection profile. The side bars represent the total frequency of occurrence of each taxon. Adapted using R Development Core Team (2013).

Co-infection (≥2 taxa) was recorded in 9 of the 19 animals (47.4%), with five individuals (26.3%) harboring three or more taxa simultaneously. The most frequent infection profiles were *D. renale* alone and absence of infection (*n* = 3 each); a profile of *Oncicola* spp. (a single taxon) was observed in two individuals. All other combinations occurred in a single host (Figure 14).

A small number of taxa accounted for most of the occurrences and, therefore, underlay most of the observed intersections. The most recurrent were *P. affinis*, *Oncicola* spp., and *D. renale*. The remaining helminths exhibited low frequency and tended to form occasional combinations. In total, 6 of the 17 identified taxa (*T. canis*, *A. braziliense*, *A. caninum*, *Ancylostoma* spp., *D. renale*, and *Spirometra* spp.) are zoonotically important. Although not currently considered a zoonosis of epidemiological relevance, species of the genus *Physaloptera* have been recorded parasitizing humans in Africa and South America [33], evidencing the occasional potential for human infection by this group of nematodes.

## 4. Discussion

We recorded 17 helminth taxa distributed across 11 genera in wild canids from three Brazilian biomes, with an overall infection occurrence of 84.2%. This richness is consistent with findings from other surveys of wild canids in the Neotropical region [34,35], in which the diversity of invertebrate intermediate hosts and the generalist feeding behavior of these carnivores favor the acquisition of helminths with indirect life cycles [36,37].

The dominance of Nematoda (80.5%) in this study, both in terms of taxonomic richness and mean abundance, reinforces the pattern observed in helminthological surveys of wild carnivores [34,35,38,39]. Furthermore, it reflects the influence of the diversity of transmission routes available to this group, including direct fecal–oral transmission, cutaneous penetration, and predation on vertebrate or invertebrate intermediate hosts [40,41].

*Cerdocyon thous* exhibited the highest helminth species richness in this study (14 taxa). This is attributable to its feeding habits and diversified habitat use [42], reinforcing its role in maintaining parasitic cycles and as an indicator of exploited biological niches. Lima et al. [35] previously documented 16 helminth species, including representatives of four classes, i.e., Nematoda, Cestoda, Trematoda, and Acanthocephala, in this host in the Caatinga biome.

This study highlights the trophic versatility of *C. thous* and its role as a definitive host for a diverse parasite community. This is evidenced by the simultaneous occurrence of helminths with direct and indirect life cycles, including nematodes transmitted via the fecal–oral or percutaneous route, such as the genera *Ancylostoma* and *Toxocara*; plerocercoid larvae of the genus *Spirometra*, which require intermediate hosts; and trematodes and acanthocephalans dependent on arthropods as intermediate hosts.

The highest parasite burden recorded in this study was observed in *C. brachyurus*, infected with *A. caninum*, with two infected individuals harboring an average of 309.5 worms each (MI 123.8). The magnitude of this infection reflects the proximity between wild and domestic environments, suggesting overlapping ecological niches and potential transmission routes between domestic and wild populations [2]. The genus *Ancylostoma* is frequently reported in domestic animals in Brazil, with high prevalence associated with favorable climatic and environmental conditions, particularly in tropical regions [43].

*Ancylostoma caninum* is a parasite that occurs predominantly in domestic canids. Its high prevalence in wild carnivores at peri-urban interfaces suggests continuous spillover transmission, associated with host interaction with human-altered landscapes. This reinforces the influence of anthropogenic activities on infection dynamics, the persistence of the parasitic cycle, and the health risks to wildlife [39,44,45,46].

*Dioctophyme renale* was detected in 60% of the *C. brachyurus* examined (*n* = 3/5), consistent with the recognized susceptibility of this canid species to infection by this nematode in Brazil [47,48]. The life cycle of *D. renale* requires the ingestion of intermediate and paratenic hosts (oligochaetes, fish, and frogs). In regions with high prevalence, the habit of drinking water from canals, as well as consuming fish or anurans, is an important risk factor for domestic and wild animals [48,49]. The occurrence recorded in this study is among the highest reported for this parasite in wild canids in Brazil. We attribute this to the sampling sites’ characteristics favorable to the development of the biological cycle, both in climatic terms and in terms of the biodiversity of definitive, intermediate, and paratenic hosts, features typical of the Cerrado biome. The finding in *C. brachyurus* and its absence in other wild canids indicate a closer trophic relationship between *C. brachyurus* and *D. renale* than with the others.

*Oncicola* spp. were recorded in all examined specimens of *S. venaticus* (Oc 100%; MI = MA = 75.5), revealing the highest occurrence and intensity values recorded for any taxon in this study. Acanthocephalans of this genus use arthropods as intermediate hosts and vertebrates as paratenic hosts [50]. The diet of *S. venaticus* predominantly comprises small mammals, birds, and reptiles [51], facilitating repeated exposure to paratenic hosts carrying cystacanths. This accounts for the infection intensity observed in this study. The small sample size (*n* = 2) precludes definitive conclusions at the population level; however, the pattern suggests the need for further investigation of this endangered species.

Additionally, specimens of *S. lupi* were identified in one individual of *S. venaticus* (Oc 50%; MI 13; MA 6.5), associated with nodular gastric lesions documented by histopathology (Section 3.3). Most *S. lupi* parasitic nodules are observed in the caudal esophagus. However, the present study identified a solitary nodule in the stomach, consistent with aberrant migration [52]. *S. lupi* has previously been reported in wild *Lycalopex culpaeus*, *Canis latrans*, *Vulpes vulpes*, and *S. venaticus* in South America [53,54,55,56,57,58]. This is the first report of *S. lupi* in the stomach of *S. venaticus* in the Brazilian Cerrado, expanding the knowledge of its distribution and potential pathological implications in wild canids. Although the cause of death was attributed to traumatic injury, the incidental discovery of *S. lupi* with associated gastric disease in *S. venaticus* raises concerns regarding the potential impact of this parasite on the health of this vulnerable species.

*P. affinis* and *A. foxi* were recorded in *L. vetulus* for the first time in this study, representing new host records for both parasites in this canid species worldwide. The helminth fauna of *L. vetulus* remains poorly studied, and the two taxa recorded herein expand the range of parasites known for this host. *P. affinis* is a nematode transmitted through the ingestion of arthropod intermediate hosts [35], with records in South American canids, including *C. thous* in Brazil (Amazônia e Caatinga) [35,59] and *Lycalopex gymnocercus* in Argentina [38]. The insectivorous diet of the genus *Lycalopex*, centered on termites and other invertebrates, favors exposure to this spirurid [42].

The occurrence of *A. foxi*, a digenean trematode with an indirect life cycle, represents an unusual finding because the family (Dicrocoeliidae) to which it belongs requires intermediate hosts such as mollusks and arthropods, and the insectivorous habits of *L. vetulus* suggest opportunistic ingestion of infected invertebrates [42]. *A. foxi* was the first species of its genus to be described as infecting mammals, having been first observed in the liver of the South American primate *Cebus capucinus* [60]. In contrast, the remaining species of the genus are predominantly associated with birds as hosts. The species, associated with hepatic and inflammatory alterations, was previously reported in the maned wolf (*C. brachyurus*) in Rio de Janeiro, Brazil, representing one of the few documented records for the family Canidae in South America [61]. Additionally, *A. foxi* was also identified in *C. thous* and *C. brachyurus* in this study, confirming the species’ capacity to infect different wild canids and evidencing its circulation in environments shared by these hosts.

Plerocercoids of *Spirometra* spp. (sparganum) were recovered from one specimen of *C. thous* in the Pantanal biome (Oc 10.0%; MI 8; MA 0.8), representing the first record of sparganosis in this host species. *Spirometra* spp. are a cestode of the family Diphyllobothriidae, with a heteroxenous life cycle in which adult worms parasitize the small intestine of carnivores, whereas the larval plerocercoid form (sparganum) lodges in the tissues of intermediate and paratenic hosts, including amphibians, reptiles, birds, and mammals [40,62,63]. The recovery of plerocercoids rather than adult tapeworms suggested that the *C. thous* specimen examined served as a paratenic host, having ingested secondary intermediate hosts (likely amphibians or reptiles) harboring infective larvae.

The presence of *Spirometra* spp. in the examined canids suggests the consumption of infected prey. It highlights the influence of predatory behavior and carnivorous diet on the transmission of this cycle in wild populations [64]. This finding is believed to be associated with the ecological particularities of the Pantanal, which is characterized by a mosaic of habitats shaped by seasonal flooding and drought cycles. This influences the availability of primary and secondary intermediate hosts (copepods, amphibians, and reptiles) involved in the biological cycle of *Spirometra* spp. [65]. The zoonotic importance of this finding should not be overlooked. In humans, the larvae can cause sparganosis, whereas infection by adult worms is rare [66].

The intensification of anthropogenic environmental changes and the narrowing of the human–animal interface favor the sharing of zoonotic parasites, reinforcing the importance of parasitological surveys in wild and human hosts from a One Health perspective [12]. In their analysis of 22 studies, Carrera-Játiva et al. [67] observed that nine (39%) reported a significant increase in helminth occurrence in small mammals from degraded or fragmented habitats compared with those from more preserved areas. The parasites most frequently associated with this pattern included mainly monoxenous nematodes, as well as some heteroxenous species, such as *Physaloptera* spp. and representatives of the class Trematoda, a pattern consistent with the results of this study.

Species of the genus *Physaloptera* are widely distributed and exhibit low host specificity, having been recorded in various domestic and wild carnivores [22,68,69,70,71,72]. In South American canids, *P. digitata*, *P. praeputialis*, and *P. terdentata* have previously been described in *C. thous*, *L. gymnocercus*, and *C. brachyurus* in Argentina [73], Bolivia [74], and Brazil [34,35], indicating a wide distribution of the genus across the continent. The records obtained in the present study reinforce this pattern and confirm the recurrence of *Physaloptera* spp. in *C. thous* in the Cerrado and Pantanal regions. Infection by this nematode in wild carnivores is associated with the consumption of arthropods that act as intermediate hosts [36,75]. Given the opportunistic and omnivorous diet of *C. thous*, occasional consumption of arthropods may be favored, particularly during periods of food scarcity. The presence of this parasite in the examined individuals confirms that trophic chains involving intermediate hosts remain functionally active in the studied environment, contributing to the maintenance of the parasitic cycle.

Wild animals play a central role in the epidemiology of most zoonoses, acting as reservoirs for the transmission of zoonotic agents to domestic animals and humans [76]. The consumption of intermediate and paratenic hosts by wild carnivores favors the maintenance and dissemination of parasites with indirect cycles, as evidenced in this study. The occurrence of helminths with different life cycles and transmission strategies reinforces the broad network of ecological interactions established by wild canids, highlighting their role in connecting natural environments, anthropized areas, and hosts of different trophic levels.

## 5. Conclusions

The identification of new parasite–host associations in this study reinforces the importance of parasitological investigations for advancing the knowledge of Brazilian biodiversity and for assessing potential ecological and epidemiological impacts. Among the identified helminths, species of zoonotic importance stand out, such as *T. canis*, *A. braziliense*, *A. caninum*, *D. renale*, *Physaloptera* spp., and *Spirometra* spp., recognized for their ability to infect humans and cause syndromes such as visceral and cutaneous larva migrans and sparganosis, in addition to rare accidental infections.

Understanding the dynamics of parasitism in the Brazilian Amazon, Pantanal, and Cerrado biomes requires an integrated analysis of interactions among parasites, hosts, and environmental factors, which may favor the interspecific transmission of zoonotic pathogens. These findings reinforce the need for continuous monitoring programs that integrate animal health, public health, and environmental conservation, aiming to mitigate the risks associated with zoonoses in ecosystems undergoing constant transformation.

The study aimed solely to present epidemiological data. The absence of molecular data might appear to be a limitation; however, new studies focusing on specific groups and species are ongoing to address the remaining gaps regarding these helminth groups.

## Figures and Tables

**Table 1:** Occurrence of parasitism in wild canids by host species and biome.

Species	Specimen	Biome	City, Province	Helminth (*n*) *	Host ID **
*Cerdocyon thous*	1	Pantanal	Poconé, MT	*Toxocara canis* (3)	CHUFJ 0095
				*Physaloptera praeputialis* (2)	CHUFJ 0096
				*Spirometra* spp. (8)	CHUFJ 0093
	2	Amazônia	Vila Bela da Santíssima Trindade, MT	*T. canis* (32)	CHUFJ 0101
				*Ancylostoma* sp. (1)	CHUFJ 0099
	3	Cerrado	Jataí, GO	*Physaloptera digitata* (8)	CHUFJ 0135
				*Pterygodermatites affinis* (17)	CHUFJ 0138
	4	Cerrado	Jataí, GO	*P. praeputialis* (19)	CHUFJ 0144
				*Physaloptera* sp. (1)	CHUFJ 0142
				*P. affinis* (14)	CHUFJ 0150
				*Pterygodermatites* spp. (6)	CHUFJ 0152
				*Oncicola* spp. (4)	CHUFJ 0153
	5	Cerrado	Jataí, GO	*Ancylostoma braziliense* (2)	CHUFJ 0124
				*P. digitata* (11)	CHUFJ 0130
				*P. praeputialis* (2)	CHUFJ 0125
				*Physaloptera terdentata* (6)	CHUFJ 0126
				*P. affinis* (4)	CHUFJ 0128
				*Pterygodermatites* spp. (14)	CHUFJ 0123
	6	Cerrado	Jataí, GO	-	-
	7	Cerrado	Jataí, GO	-	-
	8	Cerrado	Cuiabá, MT	*Oncicola* spp. (3)	CHUFJ 0102
	9	Cerrado	Cuiabá, MT	*A. braziliense* (8)	CHUFJ 0114
				*Ancylostoma* spp. (2)	CHUFJ 0115
				*P. affinis* (29)	CHUFJ 0118
				Oligacanthorhynchidae (3)	CHUFJ 0117
				*Sphaerirostris* sp. (1)	CHUFJ 0116
	10	Cerrado	Jataí, GO	*Athesmia foxi* (6)	CHUFJ 0155
*Chrysocyon brachyurus*	1	Pantanal	Poconé, MT	*Ancylostoma caninum* (605)	CHUFJ 0098
	2	Cerrado	Mineiros, GO	*A. caninum* (14)	CHUFJ 0113
				*Pterygodermatites* spp. (3)	CHUFJ 0112
				*A. foxi* (21)	CHUFJ 0110
	3	Cerrado	Jataí, GO	*D. renale* (3)	-
	4	Cerrado	Serranópolis, GO	*D. renale* (5)	-
	5	Cerrado	Mineiros, GO	*D. renale* (1)	-
*Lycalopex vetulus*	1	Cerrado	Jataí, GO	-	-
	2	Cerrado	Jataí, GO	*P. affinis* (4)	CHUFJ 0120
				*A. foxi* (5)	CHUFJ 0119
*Speothos venaticus*	1	Cerrado	Nova Mutum, MT	*T. canis* (7)	CHUFJ 0088
				*Oncicola* spp. (124)	CHUFJ 0091
	2	Cerrado	Jataí, GO	*Spirocerca lupi* (13)	CHUFJ 0105
				*Oncicola* spp. (27)	CHUFJ 0108

* Entries marked with “-” in this column correspond to negative hosts. ** Entries marked with “-” in this column correspond to specimens that could not be deposited due to a lack of specimen integrity or negative hosts.

**Table 2:** Occurrence (Oc), mean intensity (MI), and mean abundance (MA) of gastrointestinal helminths by host species in wild canids from the Cerrado, Pantanal, and Amazon biomes, Brazil.

Helminth Species	*Cerdocyon thous* (*n* = 10)			*Chrysocyon brachyurus* (*n* = 5)			*Lycalopex vetulus* (*n* = 2)			*Speothos venaticus* (*n* = 2)		
	Occurrence	MI	MA	Occurrence	MI	MA	Occurrence	MI	MA	Occurrence	MI	MA
Nematoda												
*Toxocara canis*	20	17.5	3.5	-	-	-	-	-	-	50	7	3.5
*Ancylostoma braziliense*	20	5	1	-	-	-	-	-	-	-	-	-
*Ancylostoma caninum*	-	-	-	40	309.5	123.8	-	-	-	-	-	-
*Ancylostoma* spp.	20	1.5	0.3	-	-	-	-	-	-	-	-	-
*Spirocerca lupi*	-	-	-	-	-	-	-	-	-	50	13	6.5
*Physaloptera digitata*	20	11.5	2.3	-	-	-	-	-	-	-	-	-
*Physaloptera praeputialis*	30	7.7	2.3	-	-	-	-	-	-	-	-	-
*Physaloptera terdentata*	10	6	0.6	-	-	-	-	-	-	-	-	-
*Physaloptera* spp.	10	1	0.1	-	-	-	-	-	-	-	-	-
*Pterygodermatites affinis*	40	16	6.4	-	-	-	50	4	2	-	-	-
*Pterygodermatites* spp.	20	10	2	20	3	0.6	-	-	-	-	-	-
*Dioctophyme renale*	-	-	-	60	3	1.8	-	-	-	-	-	-
Cestoda												
*Spirometra* spp.	10	8	0.8	-	-	-	-	-	-	-	-	-
Trematoda												
*Athesmia foxi*	10	3	0.6	20	21	4.2	50	5	2.5	-	-	-
Acanthocephala												
*Oncicola* spp.	20	3.5	0.7	-	-	-	-	-	-	100	75.5	75.5
Oligacanthorhynchidae	10	3	0.3	-	-	-	-	-	-	-	-	-
*Sphaerirostris* spp.	10	1	0.1	-	-	-	-	-	-	-	-	-

## Data Availability

All generated data are contained in the manuscript.
